# Effect of seed position and soil nutrients on seed mass, germination and seedling growth in *Peucedanum oreoselinum* (Apiaceae)

**DOI:** 10.1038/s41598-017-02035-1

**Published:** 2017-05-16

**Authors:** Jeremi Kołodziejek

**Affiliations:** Department of Geobotany and Plant Ecology, University of Lodz, 12/16 Banacha St., 90-237 Lodz, Poland

## Abstract

There is large variation in seed mass within *P*. *oreoselinum* (L.) Moench selected for the present study from two contrasting habitats: roadside and oak forest. Effect of seed position within a plant and of soil nutrients on seed mass, germination and seedlings growth were studied. Within an individual plant, seed mass decreased with umbel order and seeds from the central umbellet of the umbel were lighter than those from the outer edge, suggesting that variation in seed mass within an individual plant was due to the position effect. There was a significant relationship between seed mass and total germination. Covariate analysis showed the differences between sites in seed macronutrient contents were caused by respective differences in seed mass and soil macronutrients. This indicates substantial variation in the amount of reserves initially available for seedling growth. In conclusion, phenotypically-based variation in seed mass may arise from soil conditions, maternal traits or combination of the two. High variability in seed mass of *P*. *oreoselinum* favours its widespread geographic distribution. These results suggest that with respect to germination characteristics large seeds from primary order have a competitive advantage over small seeds produced on secondary umbels because they have higher overall germination.

## Introduction

Seed size (mass) can affect germination time, germination percentage, dispersal, seed water relation, as well as seedling establishment, growth, survival, and thus it has important ecological consequences^1–5^. Seed mass within an individual species was considered for a long time to be relatively constant^6^. However, various studies have demonstrated that seed size may greaten vary in a given species among sites, among plants within given site, and even an individual plant^7^. The main reasons of seed mass variations are paternal genetic effect, timing of flowering and fecundation, brood size, sibling rivalry and position effect of seeds within a plant, or in inflorescence, and the position of a seed within fruit^8^.

Variation in seed mass has often been correlated with environmental factors^7^. The contents of various nutrients in plants vary with plant species and varieties and depend on the total nutrients supply in soils and on factors controlling their availability to plants. However, studies addressing the influence of environmental factors on seed mass in natural sites are rare. An increase in the macronutrient concentration in surrounding environment has often led to the production of seeds that are heavier and have greater quantities and concentrations of N (nitrogen) and P (phosphorus)^9^. This plastic response can have important fitness consequences for developing seedlings. The positive effects of seed size are often impossible to separate from those of seed mineral content, especially in wild habitats where variations in plant characteristics are great and not easily controlled^10^.

Variation in seedling size may by caused by differences in initial seed mass, microsite characteristics and/or genotypic variation^6^. For example, it is generally accepted that seedling size is usually directly related with food reserves and energy content of seeds^6, 7^. Since the inflorescence of the Apiaceae family consists of a series of sequentially formed umbels producing seeds, the position of a seed or fruit on a plant can affect seed mass, morphology, germination and dormancy characteristics^11–14^. Positional effect on seed mass was reported widely for many species. For example, Ojala^15^ observed that seed mass and germination percentage of *Angelica archangelica* were higher in seeds on the primary umbel than in those on secondary, tertiary, and quaternary umbels. Thomas^16^ and Thomas *et al*.^17, 18^ found reduced germination of *Petroselinium crispum*, *Daucus carota* and *Apium graveolens* seeds produced on secondary, tertiary, and quaternary umbels compared with those on primary umbels. Gray and Steckel^19^ found that umbel order affected germination of *D*. *carota* seeds, but on the other hand, in *Heracleum mantegazzianum* ^13^ and in *Angelica acutiloba* ^14^ germination percentage of the seeds obtained from different umbel orders and umbellet positions did not differ significantly.

Effects of seed position within a plant and of soil nutrients on seed mass, germination and seedlings growth were studied. Seeds harvested from primary and secondary umbels were examined. The objectives of this study were the following (1) to analyze the effect of the umbel order and umbellet position on seed mass and seed germination; (2) to quantify the degree of seed mass variation in *P*. *oreoselinum* among populations and plants within populations, and within individuals plants; (3) to determine possible relation between seed macronutrient content and respective soil macronutrients content; and (4) to examine the relationship between seed mass and seed macronutrient content.

## Results

### Soil nutrients

The Student’s t-test revealed statistically significant differences in soil nutrient contents between the studied habitats. Soils at the roadside contained lower concentrations of N (Student’s t-test, p-value = 0.0025), P (Student’s t-test, p-value = 0.0309), and K (Student’s t-test, p-value = 0.0445) than those in the forest (Table 1) probably reflecting their lower content in the parent rock.

**Table 1:** Site characteristics and soil macronutrient (N, P or K) content (total N%, others mg/100 g).

Site	Coordinates	Habitat	N (%)	P (mg/100 g)	K (mg/100 g)
Lipnica	51.913690°N	roadside	0.9 ± 0.1b	7.32 ± 1.76b	4.2 ± 0.2b
	18.868497°E				
Wilczków	51.928406°N	forest	2.6 ± 0.1a	9.29 ± 1.23a	5.4 ± 0.3a
	18.897422°N				

Statistical comparisons (Student’s t-test) within a column only; different letters beside means denote significant differences between habitats at p < 0.05. Data are means (±SD) from five samples at each habitat.

### Seed mass

**Figure 1:**
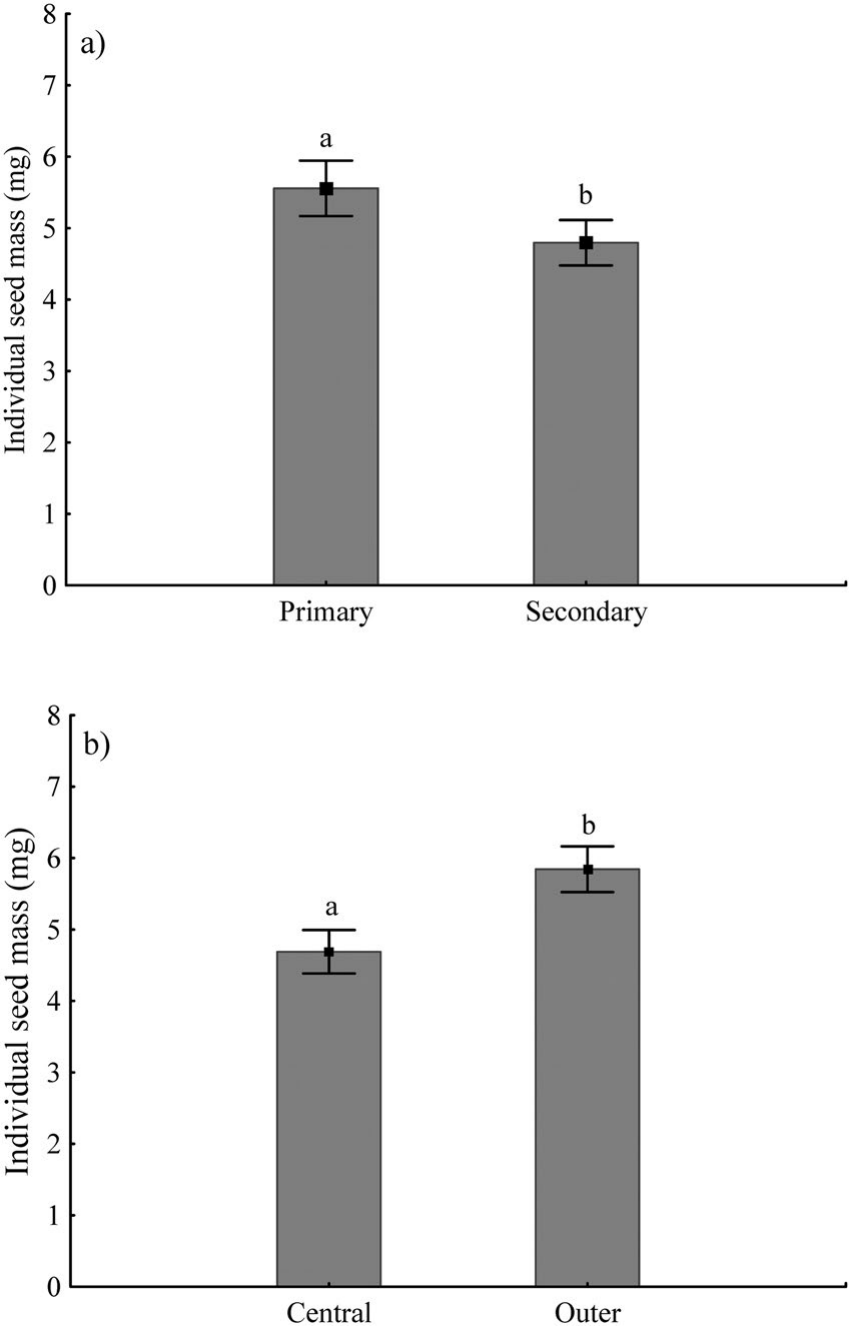
Differences in mean seed mass (±SD) between (**a**) umbel orders (primary and secondary) and (**b**) umbellet position (outer and central) in *Peucedanum oreoselinum*. Overall comparison between means was performed by Student’s t-test; different letters indicate significant (p < 0.05) differences between means.

The mean mass of 25 seeds, pooled across plants and habitats, was 134 ± 19 mg, giving the mean mass of a single seed of 5.36 mg. Seed mass was variable: 1.7 fold between habitats, 1.6–1.9 fold within habitats, 1.4–1.6 fold within individuals, and 1.2–1.4 fold within umbels. Coefficients of variation between habitats averaged 14% (range 13–15) (Table 2). There was great difference in mean seed mass between roadside and forest (3.7 ± 0.3 mg and 6.9 ± 0.2 mg, respectively). The mean seed mass within a plant decreased significantly (p-value = 0.0241) from primary to secondary umbels in both habitats. Seeds produced by the primary umbels were significantly (p-value = 0.0217) heavier (5.8 ± 0.3 mg) than those from the secondary ones (4.8 ± 0.4 mg) (Fig. 1a). Seed mass was also very strongly influenced by the position within an umbel: the seeds from the outer edge of the umbel were heavier (5.9 ± 0.4 mg) than those from the center of the umbel (4.7 ± 0.3 mg) (Fig. 1b). Nested analysis of variance indicated that the percentage of variation in seed mass accounted for by plants and by umbels within plants was 25% and 21%, respectively (Table 3).

**Table 2:** Variations in seed mass within sites, individuals, and umbel order.

Habitat	Within habitat			Within plants	Within primary order	Within secondary order
	Range in seed mass (mg)	Magnitude in individual seed mass variation (heaviest/lightest)	CV (%)	Mean seed mass variation (heaviest/lightest)	Mean seed mass variation (heaviest/lightest)	Mean seed mass variation (heaviest/lightest)
Roadside	3.5–6.5	1.9	15.4	1.6	1.2	1.3
Forest	4.4–7.2	1.6	13.0	1.4	1.4	1.2
Total	4.0–6.9	1.7	14.2			

For the determination of average seed mass four replicates of 25 seeds each were weighed.

**Table 3:** Results of three-level nested analysis of variance testing for effect of habitat, individuals, and umbels on seed mass in *Peucedanum oreoselinum*.

Source of variation	df	MS	Variance component	Proportion of variance explained
Habitat	1	14.67***	0.034	29.1
Plants within habitat	126	0.56**	0.012	25.6
Umbels within plants	967	0.14**	0.019	21.2
Error (within umbels)	8872	0.03***	0.021	24.1
Total	9978		0.086	100

***p < 0.001, **p < 0.001 (Tukey’s test).

### Effect of seed source on seed germination

Seeds collected from the forest plants germinated more rapidly than those from the roadside. In addition, the total percentage of germination differed significantly (p < 0.006) between habitats. A significantly greater (p-value = 0.0231) total percentage germination was observed for those seeds from forest compared to seeds from roadside (Table 4). ANOVAs (log-transformed data) revealed that umbel order, umbellet position, habitat, and their interaction were all of significant importance to both seed mass and percentage of germination, but there was no significant interaction effect on rate of germination (Table 5).

**Table 4:** Seed mass, germination percentages and rate of germination (Timson’s Index) of seeds from differed umbel (primary and secondary) and umbellet position (outer and central).

Habitat	Umbel order	Umbellet position	Individual seed mass (mg)*	Final germination (%)*	Rate of germination (%)*
Roadside	Primary	Outer	5.6 ± 0.3a	81 ± 3a	58 ± 3a
		Central	4.6 ± 0.2b	64 ± 4b	56 ± 2a
	Secondary	Outer	4.9 ± 0.2ab	66 ± 3b	55 ± 4a
		Central	3.7 ± 0.3c	53 ± 5c	52 ± 3a
Forest	Primary	Outer	6.9 ± 0.2a	86 ± 3a	66 ± 6a
		Central	5.1 ± 0.5c	69 ± 5b	65 ± 4a
	Secondary	Outer	5.9 ± 0.5b	79 ± 6b	67 ± 4a
		Central	5.0 ± 0.4c	67 ± 2c	64 ± 5a

Germination data (mean ± SD) are from laboratory trials at 22 °C/10 °C with 14/10 h light treatment.

*The values followed by the same letter within a column do not differ significantly by ANOVA followed by Tukey’s test at p < 0.05 in the same habitat.

**Table 5:** Tree-way ANOVA of effect of umbel order (primary vs secondary), umbellet position (central vs outer) in the umbel, habitat (roadside vs forest), and their interaction on seed mass, percentage of germination and rate of germination of *Peucedanum oreoselinum*.

Dependent variable	Factor	df	MS	F-value	P-value
Seed mass	Umbel order (UO)	1	0.76	12.34	p = 0.0186
	Umbellet position (UP)	1	0.85	10.56	p = 0.0348
	Habitat (H)	1	0.64	7.98	p = 0.0056
	UO × UP	1	1.87	5.78	p = 0.0078
	UO × H	1	0.32	11.34	p = 0.0095
	UP × H	1	0.36	7.94	p = 0.018
	UO × UP × H	1	7.78	10.43	p = 0.0134
Percentage germination	Umbel order (UO)	1	0.34	1.23	p = 0.1266
	Umbellet position (UP)	1	1.28	1.07	p = 0.1935
	Habitat (H)	1	0.72	4.32	p = 0.0315
	UO × UP	1	1.68	12.34	p = 0.0278
	UO × H	1	0.98	4.56	p = 0.6546
	UP × H	1	1.86	5.99	p = 0.3073
	UO × UP × H	1	3.34	9.67	p = 0.0348
Rate of germination	Umbel order (UO)	1	0.57	36.93	p = 0.2605
	Umbellet position (UP)	1	1.56	34.12	p = 0.3225
	Habitat (H)	1	0.27	3.81	p = 0.0296
	UO × UP	1	2.48	17.98	p = 0.0757
	UO × H	1	0.65	2.89	p = 0.1427
	UP × H	1	1.26	1.89	p = 0.3038
	UO × UP × H	1	3.65	11.45	p = 0.0412

### Seedling growth

Overall, both shoot biomass (hereafter referred to as SB) and root biomass (hereafter referred to as RB) of the seedling from the seeds on the primary umbel were significantly higher than those of the seedlings from seeds on the secondary umbels (Table 6). These results suggests that the seedlings from seeds on the primary umbels were significantly more vigorous than those from seeds on the secondary umbels. Individual effects of umbel order, habitat, and their interaction on seedlings traits were significant. However, umbellet positions and interaction between umbel order and umbellet position had no effect on seedlings traits (Table 7). These results suggests that the seedlings from seeds from the primary umbels were more vigorous than those from the secondary umbels.

**Table 6:** Shoot biomass (SB), root biomass (RB) and the percentage of biomass allocated to root (BAR) of seedlings of *Peucedanum oreoselinum*.

Habitat	Umbel order	Umbellet position	SB (mg plant^−1^)	RB (mg plant^−1^)	BAR (%)
Roadside	Primary	Outer	13.1 ± 0.7a	4.3 ± 0.3a	24.7 ± 0.5a
		Central	12.4 ± 0.5a	4.1 ± 0.2a	24.8 ± 0.6a
	Secondary	Outer	9.0 ± 0.4b	2.7 ± 0.5b	23.1 ± 0.9b
		Central	8.8 ± 0.3b	2.6 ± 0.4b	22.8 ± 0.8b
Forest	Primary	Outer	16.1 ± 0.6a	5.9 ± 0.4a	26.8 ± 0.6a
		Central	15.7 ± 0.5a	5.8 ± 0.5a	27.0 ± 0.7a
	Secondary	Outer	13.1 ± 0.5b	3.0 ± 0.2b	18.6 ± 0.3b
		Central	13.0 ± 0.3b	2.9 ± 0.1b	18.2 ± 0.4b

Values are means ± SD.

*The values followed by the same letter within a column do not differ significantly by ANOVA followed by Tukey’s test at p < 0.05 in the same habitat.

**Table 7:** Tree-way ANOVA of effect of umbel order (primary vs secondary), umbellet position (central vs outer) in the umbel, habitat (roadside vs forest), and their interaction on seedling traits (shoot biomass [SB], root biomass [RB], and the percentage of biomass allocated to root [(BAR]) of *Peucedanum oreoselinum*.

Dependent variable	Factor	df	MS	F-value	p-value
SB (mg plant^−1^)	Umbel order (UO)	1	1.68	14.56	p = 0.0255
	Umbellet position (UP)	1	1.98	18.56	p = 0.1267
	Habitat (H)	1	1.46	5.45	p = 0.0079
	UO × UP	1	1.87	15.78	p = 0.0786
	UO × H	1	1.23	2.56	P = 0.0353
	UP × H	1	8.70	11.56	p = 0.0155
	UO × UP × H	1	7.78	10.43	p = 0.0134
RB (mg plant^−1^)	Umbel order (UO)	1	1.65	1.76	p = 0.0052
	Umbellet position (UP)	1	3.08	12.02	p = 0.2344
	Habitat (H)	1	1.34	3.98	p = 0.0046
	UO × UP	1	1.87	15.34	p = 0.0978
	UO × H	1	1.59	6.86	p = 0.0042
	UP × H	1	8.60	5.99	p = 0.0307
	UO × UP × H	1	3.34	9.67	p = 0.0348
BAR (%)	Umbel order (UO)	1	1.23	12.54	p = 0.0077
	Umbellet position (UP)	1	3.08	12.02	p = 0.2347
	Habitat (H)	1	1.51	4.32	p = 0.0026
	UO × UP	1	2.48	17.98	p = 0.0657
	UO × H	1	1.63	3.27	p = 0.0344
	UP × H	1	8.60	5.99	p = 0.0307
	UO × UP × H	1	3.65	11.45	p = 0.0412

### Seed nutrient analyses

**Figure 2:**
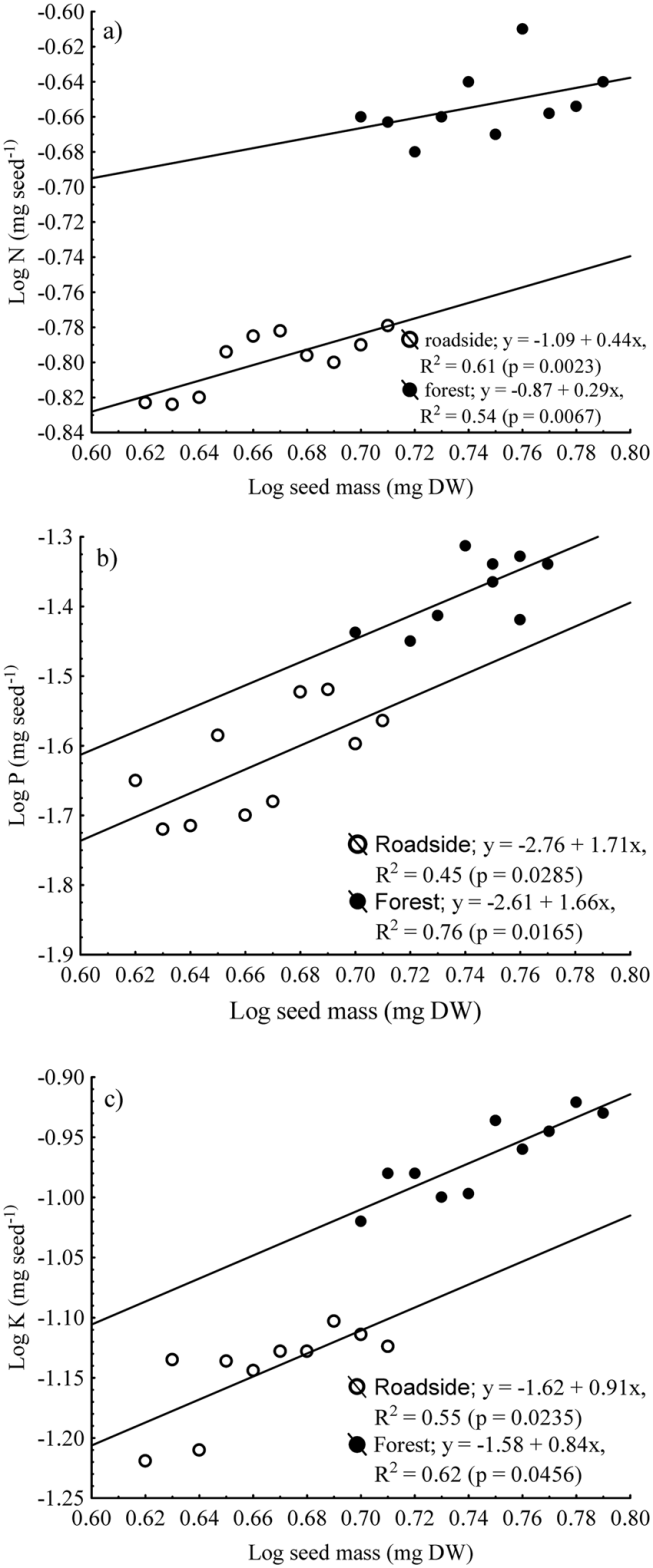
Relationships between seed mass (mg dry wt) and the (**a**) N, (**b**) P and (**c**) K content (mg dry wt seed^−1^) of seeds. Least-squares linear regression fitted to log_10_ transformed variables (solid lines). The coefficient of determination (R^2^) and the significance of the coefficient (P) are provided (n = 5). See Table 8 for analyses.

For all seed macronutrients, there were differences between habitats. The mean N, P, or K contents in seeds were significantly greater (N, p-value = 0.0234; P, p-value = 0.0365; K, p-value = 0.0216) for the forest than roadside seeds. Relationships between seed mass and seed nutrient contents differed between habitats, as indicated by significant habitat × seed mass interactions (Table 8). No significant differences were found for seed N:P ratio between habitats. The N, P and K contents of seeds (Fig. 2a–c) increased linearly with seed mass, as indicated by the slope (*b*) of the regression functions. The linear relationships between seed mass and seed content indicated that the increase in seed content per unit of seed mass was relatively uniform across seeds.

**Table 8:** ANCOVAs of the effect of seed mass (covariate) and habitat (roadside vs forest) on the nitrogen (N), phosphorus (P) or potassium (K) content in seeds based on the data pooled across umbel orders.

Nutrient	Seed mass	Habitat	Habitat × seed mass	Roadside (mg seed^−1^)	Forest (mg seed^−1^)
Nitrogen	2789.28***	6.56*	7.01*	0.16 ± 0.004a	0.21 ± 0.008b
Phosphorus	1356.14***	5.44*	6.12*	0.03 ± 0.004a	0.04 ± 0.004b
Potassium	342.31***	6.34*	5.98*	0.07 ± 0.005a	0.12 ± 0.006b
N:P	45.32^ns^	1.45^ns^	3.32^ns^	5.33 ± 0.24	5.25 ± 0.31

Total reserves of seeds (N, P or K mg seed^−1^) were calculated from the mass (mg dry weight [dry wt]) of each seed multiplied by the relative N, P or K concentration (% dry wt). Individual seed mass was obtained by taking the mean mass of sample of 100 seeds (N = 10 samples).

F-values for seed mass, habitat, and habitat × seed mass are given. *p < 0.05, **p < 0.01, ***p < 0.05. The values for seed mass followed by different letter in the same row are significantly different at p < 0.05 according Student’s t-test procedure.

**Figure 3:**
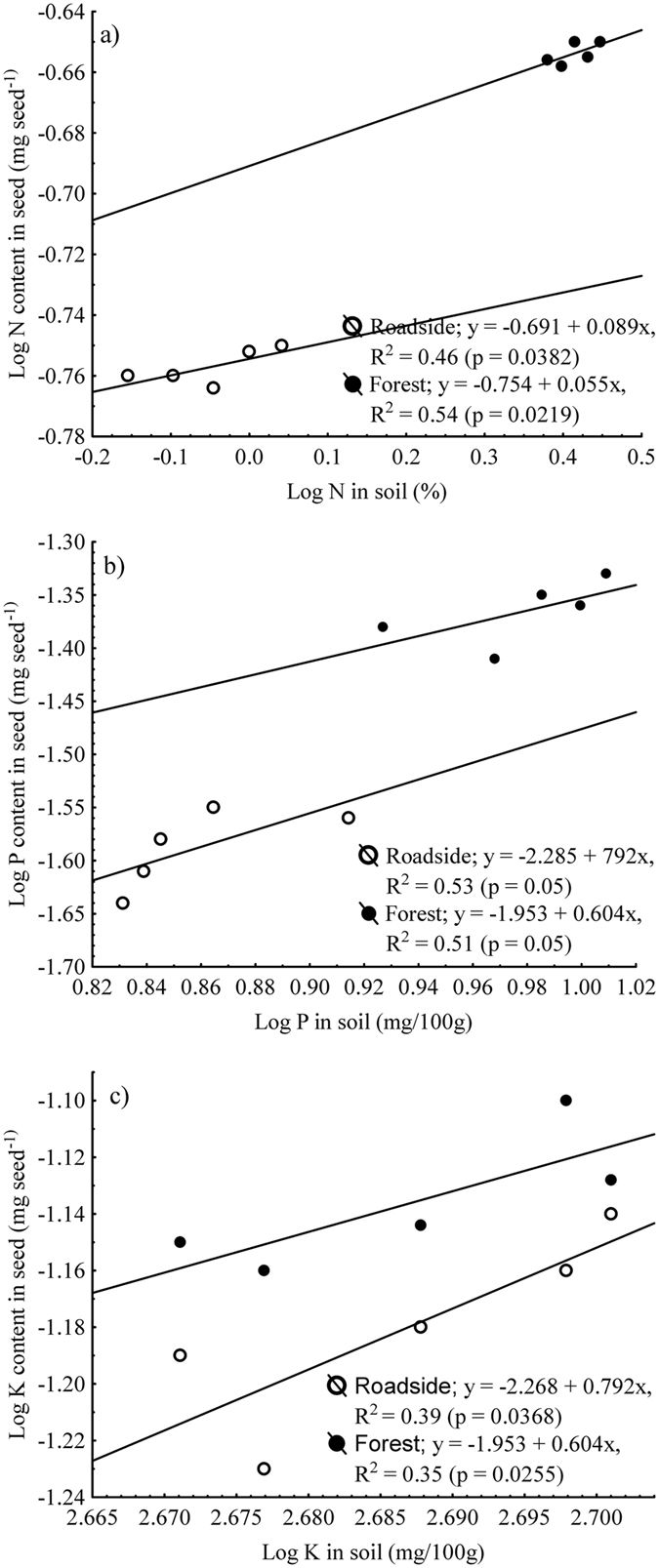
Regressions of macronutrient contents of seeds (*y*) on respective soil macronutrients (*x*): (**a**) nitrogen, (**b**) phosphorus and (**c**) potassium. Each point is the mean of 5 replicates of soils.

Regression of seed N, P, and K contents shows that seed N, P and K contents were positively related to soil macronutrients contents in each habitat (Fig. 3a–c).

## Discussion

In *P*. *oreoselinum* the seeds developed in the primary umbels were heavier than those produced by secondary umbels. In *Lomatium grayi*, 16% of the variance in seed mass within individual plants was explained by differences between umbels^20^.

The mean mass individual seed produced in forest site was striking by 2-fold greater than that in roadside site. A substantial portion of this variation is likely to have been the results of soil fertility. Soils at the roadside contained significantly lower concentrations of macronutrients than those in the forest; hence the levels of these nutrients in the seeds were different depending on the growth site. It is also likely that there is genetic differentiation between population for seed size. A similar result was already found for *Stellaria media* by Sobey^21^ and for *Senecio jacobaea* by Leiss and Müller-Schärer^22^.

This study also documents that the mass of the primary seeds was slightly more than twice that of the secondary seeds. This is consistent with the degree of variation reported for *P*. *sativa* ^5^. These differences are thought to be due mainly to variations in resources available to individual seeds. Hendrix^5^ suggested that the production of smaller seeds on later-developing umbels (e.g. secondary umbels) might be due to the decrease in the amounts of available resources over the growth season, which is disadvantageous for later-developing umbels as they compete for limited resources.

Variation of seed mass within umbels of *P*. *oreoselinum* was correlated with position: seeds from the outer edges of the umbels were heavier than those from their center. However, for seeds of *Heracleum mantegazzianum* the situation was opposite^13^, while for *Angelica acutiloba* no such differences were observed^14^.

A significantly greater total percentage germination was observed for those seeds from forest compared to seeds from roadside. Variations in seed germinability between different plant populations of a given species were previously documented. For example, Milberg *et al*.^23^ reported such a situation in *Lithospermum arvense* and *Anchusa arvensis*.

The present data show that the seed germination percentages were significantly different between the two umbel orders. This resulted from the fact that the seeds produced on the primary umbels were heavier. A positive relationship between seed mass and germination percentage has been found in *Daucus carota* ^19^, *P*. *sativa* ^5^, and *Angelica acutiloba* ^14^. These results are compatible with the view that rapid and better germination of seeds would result from larger food reserves and energy content in heavier seeds^6^. However, Hendrix^5^ reported a reversed situation in *P*. *sativa*: smaller seeds germinated more rapidly than larger ones.

Within *P*. *oreoselinum* there is considerable variation in mean seed mass between habitats. This difference might be caused by environmental factors. Several studies reported that an increase in the nutrient concentration in soil often leads to the production of heavier seeds that contain higher levels of mineral nutrients^9^. Plants of *P*. *oreoselinum* from the nutrient-rich habitat (oak forest) had heavier seeds compared to the nutrient-poor one (roadside). The relationship between seed mass and soil nutrient status has received little attention. In this study, regression of seed N, P, and K contents in *P*. *oreoselinum* in relation to the levels of respective elements in soil showed that seed and soil macronutrient levels were positively correlated. Similar relationships between seed mass, seed nutrient content, and soil fertility were reported between closely related species pairs in the family Proteaceae^24^. Aarssen and Burton^25^ observed the same effect in seeds of *Senecio vulgaris*. Plants of *Senecio vulgaris* were fertilized with high, medium and low levels of NPK (20–20–20), and the resulting seeds decreased in mass with the decrease in nutrients. Mass of individual *Erigeron annuus* achenes was 24.6 and 28.6 μg for plants that were grown under low and high levels of nutrients, respectively^26^. On the other hand, Lee and Fenner^27^ found that species in the grass genus *Chionochloa* from infertile soils had larger seeds with more seed nutrients but produced seedlings with smaller shoots than those from fertile sites. The availability of seed nutrient can have important consequences for the seedling vigor. Large seeds with greater amount of mineral nutrients tend to produce more vigorous seedlings when compared to small seeds with low levels of nutrients.

An increase in the nutrient concentration of the growing environment often leads to the production of heavier seeds that contain higher levels of mineral nutrients^9^. Thus, seedlings originating from larger seeds have both more time for development and nutrients available for growth. The results observed in this study support the conclusion that larger seeds produce seedlings with larger initial size as reported by others authors^7, 9, 28^. In this scenario it would be reasonable to think that larger seeds/seedlings have higher competitive ability relative to small seeds^1, 4, 29^. This is in contrast with other studies that detected negative^30^ or no relationship^31^ between seed size.

As noted above, for all macronutrients, there were significant differences between sites, and the N, P, and K contents of the seeds were 3%, 17%, and 4% greater for the forest than roadside seeds. For both habitats seed N:P ratios slightly exceed 5. The average seed N:P ratios reported for wild herbaceous plants is 1.5–15. Nitrogen and phosphorus concentrations in plant biomass, [N] and [P], are determined by the balance of N and P uptake, C assimilation, and the losses of N and P through turnover, leaching, exudation, herbivores and parasites. In an individual plant N:P ratio may depend additionally on internal nutrient translocation^32^. When comparing plants of the same species growing at different rates, Matzek and Vitousek^33^ found that faster-growing plants did not have consistently lower N:P ratios.

In summary, the results of this study indicated that umbel order and umbellet position had significant effect on the seed mass. In addition, mineral nutrient (N, P and K) contents in the seeds varied with habitat and depended on the total nutrient contents in soils and on factors controlling their availability to plants. Thus, phenotypically-based variation in seed mass may arise from variations in soil fertility or from a combination of environmental and maternal effect^7^. In the study reported here, it was also observed also that the umbel order affected the growth of the seedlings. The primary umbel produced the heaviest seeds and subsequently the most vigorous seedlings.

## Methods

### Studied plant

**Figure 4:**
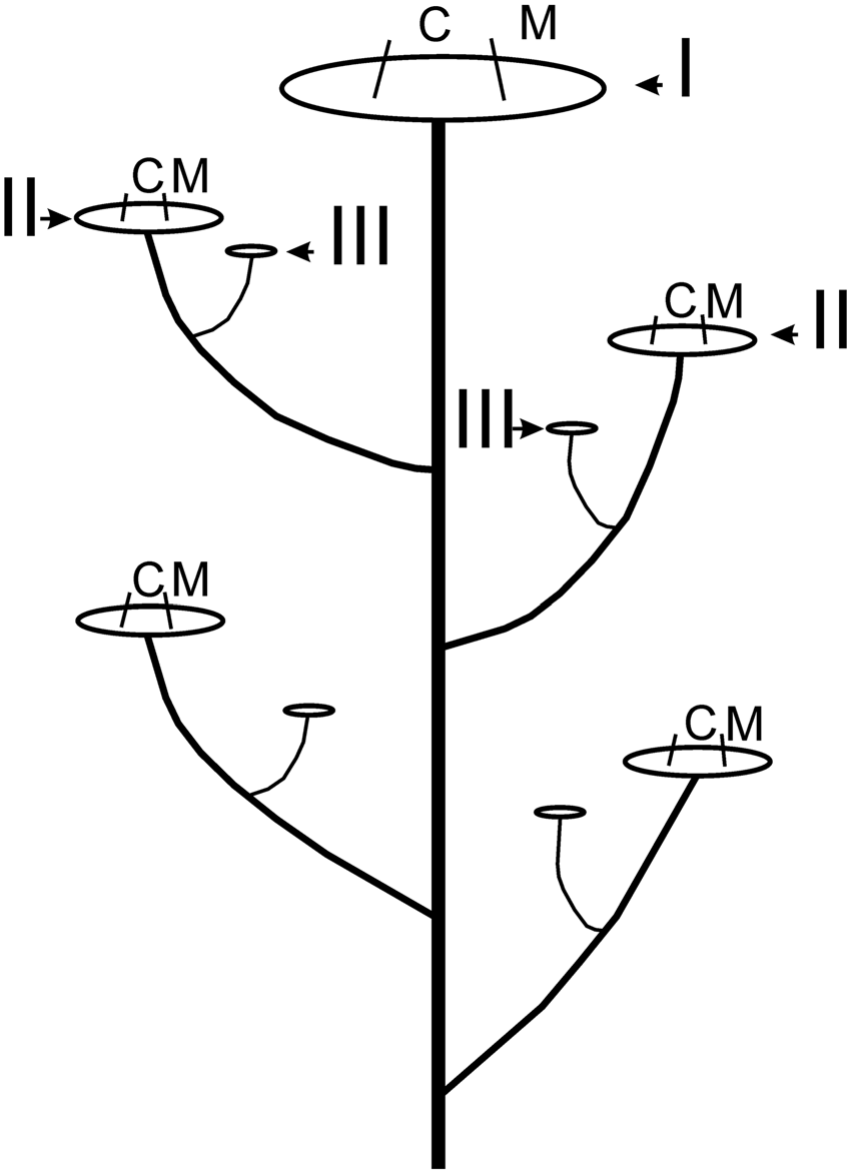
Diagram of three orders of umbels (I–III) of *Peucedanum oreoselinum*: the large first-order umbel (I), the succeeding second-order umbels (II) and the small third-order umbels (III).

*Peucedanum oreoselinum* (L.) Moench, a member of the Apiaceae, is a temperate climate species, occurring in Europe, except for the islands. It is a perennial aromatic herb growing to 1.5 m. Lower leaves are up to 40 cm, 2- to 3-pinnate, triangular in outline; upper cauline leaves less are divided. Flowers are arranged in inflorescences - compound umbels of three orders. *Peucedanum oreoselinum* grows as an erect plant^34^. The monopodial main axis ends in a terminal primary (first-order) compound umbel. Branches, which are produced on the stem, terminate in secondary (second-order) umbels. Third-order umbels may arise on shoots branching from secondary shoots (Fig. 4). Third-order umbels usually consist only of male flowers and do not produce seeds. Umbels of the first order show the greatest number of umbellets and in the umbels of progressively higher order there is a gradual reduction in the number of umbellets. The plant is in flower from June to August, and the seeds ripen from July to September. As in most species of Apiaceae, *P*. *oreoselinum* is andromonoecious (male and hermaphrodite flowers on the same plant); flowers are pollinated by insects. Petals are white or pinkish, papillose. The fruit is 5–8 mm, broadly obovate. In this paper, the fruits of the *P*. *oreoselinum* are referred to as ‘seeds’ because they are functionally analogous to true seeds. Seeds ripen in late June through July and dispers slowly. This species is commonly found on sandy, loamy soils in mildly shaded sites such as road verges, in lowland coniferous forests and deciduous forests, but also in more open places such as meadows and riverbanks^34^.

### Study sites

Two sites of *P*. *oreoselinum* were used. One site is located in Lipnica village, central Poland. At this site *P*. *oreoselinum* grow along roadside in the vicinity of agricultural fields. The other site is located near Wilczków city, 8 km from the Lipnica site. At this site, *P*. *oreoselinum* grow in the understory of an oak forest. The dominant tree is *Quercus robur* contributing 90% to the total ground cover. The undergrowth is dominated by *Convallaria majalis* which account for up to 40% of the ground cover. The following species were also recorded: *Lathyrus niger*, *Melica nutans*, *Polygonatum odoratum*, and *Vicia cassubica*. Total ground cover is ***c***. 80%. The road verge site was significantly more arid than the forest site, all the plants grew in full sunlight. The site is characterized by the following combination of species: *Alyssum alyssoides*, *Chondrilla juncea*, *Centaurea stoebe*, *Oenothera biennuis*, *Potentilla argentea*, and *Rubus plicatus*. In August 2016, seeds were collected from randomly chosen mature large plants of similar size (basal stem diameter of the flowerings talk >5 mm) growing in each of two sites in central Poland, as summarized in Table 1. In each sampled individual (N = 10) the seeds were collected from the primary and secondary umbels.

### Soil properties

In each location 5 core soil samples (2 cm diameter and 5 cm depth) were taken at random and put them in plastic bags. The samples were air-dried at 25 °C for 3 days and sieved through 2 mm mesh. The following parameters were determined in the soil samples (10 g dry soil): total nitrogen (N), phosphorus (P) and potassium (K) forms. Phosphorus was extracted with 0.5 sodium bicarbonate (NaHCO_3_), pH 8.5. and was measured colorimetrically by the molybdenum blue method. Potassium was extracted from soil with calcium lactate (C_6_H_10_CaO_6_) and determined with an atomic absorption spectrophotometer. Total nitrogen (N) was determined by the micro-Kjeldahl method by the wet oxidation of organic matter using sulfuric acid (H_2_SO_4_).

### Plant material

Samples of >150 seeds were collected from each of 10 different *P*. *oreoselinum* plants separately in two natural sites. From each plant, the seeds were harvested from four different position. Two umbellet positions (central and outer termed) within each umbel order (primary and secondary) were selected (Fig. 4). The secondary umbels chosen for seed collection were 2^nd^ from the top. The harvested seeds were air dried for 2 weeks at room temperature (±22 °C), Mean seed mass was determined by weighing 25 seeds (to the nearest 0.1 mg) per seed sample and dividing the obtained mass by 25.

### Germination test

Large-scale screening studies revealed that many temperate-climate Apiaceae species have a chilling requirement^12^, so freshly matured seeds were not tested because they have poor germination. Experimental design consisted of four variants treatments (two umbel orders × two umbellet position) performed on ten different plants from two sites. There were four replicates of each treatment × plant × site combination, giving 80 samples of seeds (=4 × 10 × 2), each consisting of 25 randomly selected seeds. The seeds were placed on filter paper, wrapped in aluminium foil and placed in a refrigerator in the dark for 8 weeks at ±5 °C. This temperature is near-optimal for many seeds requiring low moisture and low temperature to break dormancy^12^. Following stratification treatment, seeds were allowed to imbibe for 24 hours in distilled water at room temperature (±22 °C). 9-cm diameter plastic Petri dishes were lined with two filter paper discs moistened with distilled water until saturated and 25 randomly chosen seeds were distributed in each dish. The seeds were incubated at 22/10 °C in light (14 h-photoperiod, 3.000 lux, Philips 35 W/33 lamps). Moisture was maintained with deionized water. Germinated seeds were counted and removed from the Petri dishes daily. Germination was defined as germinated seeds having at least 2 mm long radicle. After 23 days, the seeds were no longer germinating, so all germinated seedlings were removed. The rate of germination was estimated using a modified Timson’s index of germination velocity:

$${\rm{G}}/{\rm{t}}$$

where *G* is seed germination percentage each day and *t* the total germination period^35^. Therefore if all of the seeds germinated in one day, the Timson’s index would be 100 (i.e., 2300/23). A higher value indicates more rapid germination.

### Seedling growth

Following stratification, a total of 800 seeds, representing 25-seed replicates of two umbel orders × two umbellet position from different four plants of two sites, were randomly selected. The seeds were then imbibed in 9-cm Petri dishes lined with 2 layers of No. 1 Whatman filter paper moistened with 3 ml distilled water. The Petri dishes (each containing 25 seeds) were placed in growth room for 3 weeks. The germination conditions were the same as those described above in germination tests. At each day of incubation, 2 ml water was added to each Petri dish. As soon as the radicle appeared the newly germinated seedlings were transferred to plastic trays (20 × 15 cm) with standard soil, consisting of a substrate of 30% sand, 20% perlite, and 50% commercial peat. Each tray containing 25 seedlings served as replicate. The trays were placed in the growth room for 4 weeks. Deionized water was added as needed. The incubation conditions were the same as those described above. By the end of the cultivation period soil was washed from the roots. Then the shoots and roots of each seedling were dried at 70 °C for 48 h to quantify shoot biomass (SB) and root biomass (RB). The percentage of biomass allocated to root (BAR)^30^ was estimated using the following formula:

$${\rm{RB}}/({\rm{RB}}+{\rm{SB}})\times 100$$

### Seed macronutrient analysis

Ten samples of 100 seeds (10 seeds from each of 10 randomly chosen plants) was used for each site. The harvested seeds were pooled from different umbel order of each individual plant and mixed. The seeds were weighed and an average mass of a individual seed for each sample was calculated. Since the nutrient contents were expressed on a dry matter basis the samples at 105 °C for 5 h. The seed material was mineralized in a boiling mixture of 10 ml of HNO_3_ and 5 ml of H_2_SO_4_. Phosphorus was determined colourimetrically (see soil properties), potassium by atomic absorption spectrometry (as above), and the total nitrogen content by the micro-Kjeldahl procedure (as above). N:P ratio was calculated from these results as N/P.

### Data analysis

The data for all statistical tests were log_10_ transformed before statistical analysis to ensure homogeneity of variance but non transformed data are shown in all figures and in tables. The data were tested for normality with the Kolmogorov-Smirnov test with the Lilliefors correction and homogeneity of variance with the Brown-Forsythe’s test. To examine variation in seed mass the coefficient of variation (CV = standard deviation/mean × 100) for seed dry mass was calculated. Mean seed mass variation was determined by dividing mass of the heaviest by mass of the lightest seeds. Significant differences in the soil macronutrient content and differences in the seed macronutrient content from different habitats were estimated using all-pair Student’s t-test comparisons. Student’s t-test was also performed to test for statistical differences in mean seed mass between umbel orders (primary vs secondary) and between umbellet position (outer vs central). One-way ANOVAs followed by Tukey’s post-hoc comparison tests were used to test for differences in seed mass, germination percentages and rate of germination (Timson’s Index) of seeds from differed umbels and umbellet positions collected for each habitat. One-way ANOVAs were also performed to test for differences in shoot biomass, root biomass and the percentage of biomass allocated to root. ANOVA was used to analyze the effect of umbel order, umbellet position in the umbel, habitat and their interaction on seedling traits (shoot biomass [SB], root biomass [RB], and the percentage of biomass allocated to root ([BAR]).

Analyses of covariance (ANCOVA) was performed to test the effect of seed mass (covariate) and habitat (roadside vs forest) on the nitrogen, phosphorus, and potassium content of seeds. Also ANCOVA was performed with seed mass as the covariate to test for significant differences between umbellet position with respect to the seedling traits indicated above. The effect of habitat, individuals, and umbels on the percentage of variation in seed mass was analyzed using a three-way nested ANOVA.

Regression analysis was carried out to assess the influence of the macronutrient content in the soil on their concentration in seed. The influence of seed mass on seed N, P or K content was also examined by lest-squares linear regression analyses. All statistical analyses were done using the Statistica 12.0 package^36^.
